# Prostatic Stones: Evidence of a Specific Chemistry Related to Infection and Presence of Bacterial Imprints

**DOI:** 10.1371/journal.pone.0051691

**Published:** 2012-12-13

**Authors:** Arnaud Dessombz, Paul Méria, Dominique Bazin, Michel Daudon

**Affiliations:** 1 Laboratoire de Physique des Solides, Université Paris Sud, Centre National de la Recherche Scientifique, Orsay, France; 2 Service d’Urologie, Hôpital Saint-Louis, Assistance Publique-Hôpitaux de Paris, Paris, France; 3 Laboratoire de la Chimie de la Matière Condensée de Paris, Université Pierre et Marie Curie, Collège de France, Centre National de la Recherche Scientifique, Paris, France; 4 Service d’Explorations Fonctionnelles, Hôpital Tenon, Assistance Publique-Hôpitaux de Paris, Paris, France; Universidade de Sao Paulo, Brazil

## Abstract

Prostatic stones are a common condition in older men in industrialized countries. However, aging appears not to be the unique pathogenesis of these calcifications. Our morpho-constitutional investigation of 23 stone samples suggested that infection has a significant role in the lithogenic process of prostate calcifications, even without detection of infection by clinical investigation. Most stones (83%) showed bacterial imprints and/or chemical composition, suggestive of a long-term infection process. Chronic infection may induce persistent inflammation of the tissue and secondarily, a cancerization process within a few years. Thus, the discovery of prostate calcifications by computerized tomodensitometry, for example, might warrant further investigation and management to search for chronic infection of the prostate gland.

## Introduction

Prostate cancer is the second most frequent cause of mortality due to cancer in males in the United States [1]. Transuretral resection, radical prostatectomy, radiation therapy and hormone therapy are the usual prostate cancer treatments [2]. Prostate removal leads to the observation of prostatic calculi. Because almost 99% of surgically removed prostates contain stones, these stones are generally considered clinically insignificant [3]. Therefore, only a few papers have investigated these calcifications.

We noted the large chemical diversity of these prostatic stones. Carbonated calcium phosphate apatite (carbapatite; CA) seems to be the major component, but several investigations show the presence of other mineral phases such as calcium oxalate monohydrate and dihydrate [4], brushite [5], and whitlockite [6]. More recently, several other mineral phases previously not reported in prostatic calculi were octacalcium phosphate pentahydrate and amorphous carbonated calcium phosphate [7]. Such chemical diversity indicates significant variations in the local biochemistry, which may be linked to different conditions.

A previous investigation involving stone culture revealed that infected calculi in the prostate were implicated in relapsing urinary tract infection [6]. This work aimed to assess a possible relationship between infection and prostatic calculi, taking into account the chemical and structural characteristics of such calculi. We combined chemical analysis with Fourier transform infra-red (FTIR) spectroscopy and structural investigation at the mesoscopic scale by scanning electron microscopy (SEM). FTIR spectroscopy has helped us to examine the presence of chemical phases involved in infection of other organs [8], [9]. Moreover, SEM observations allowed for assessing the presence of bacterial imprints on prostatic calculi [10]. FTIR spectroscopy and SEM have been used in several studies of pathological calcifications [11], [12], [13], [14], [15].

## Materials and Methods

### Samples

We investigated 23 prostatic stones obtained from the Saint-Louis and Tenon hospitals in Paris. The calcifications were collected from the prostate after radical prostatectomy or transuretral resection. The mean age of the patients was 71 years (range 35 to 87 years). All participants gave their verbal consent for use of the material. Samples were examined without knowledge of the name of the patient or other identifying data. Ethical approval for the study was obtained from the ethics committee of Tenon Hospital.

### FTIR Spectroscopy

The FTIR spectroscopy was performed at Tenon Hospital. Each sample was analysed in absorbance mode on a Bruker Vector 22 spectrometer by accumulation of 32 spectra between 4000 and 400 cm^−1^, with resolution 4 cm^−1^ and time acquisition 1 sec/spectrum. The analysis was as previously described [16]. For each sample, the inner and surface compositions were established. The compounds were identified by comparing them to reference spectra [17].

### SEM

Each prostatic stone was observed by Field-effect SEM (Zeiss SUPRA55-VP with an Everhart-Thornley secondary electron detector). To maintain sample integrity, each measurement was taken at low voltage (≤2 keV). Stones were imaged at similar magnification for comparison.

**Table 1:** Major phases in inner and peripheral layers (minor phases in italics) in 23 samples of prostate stones, along with presence of urinary infection and bacterial imprints.

N°	Age	Core	Periphery	Urinary infection	Bacterial imprints
1	81	WK>ACCP>*CA>PROT*	CA>OCP>*ACCP>PROT*	ND	Yes
2	68	CA>ACCP>>*MAP, COM, PROT*	CA> = ACCP>>*PROT*	ND	Yes
3	78	CA>>COD>*PROT>COM*	CA>>*COD, PROT*	Yes	No
4	78	CA>>COD>*COM>>MAP,PROT*	CA> = PROT>>*COD*	No	Yes
5	79	CA>WK>*PROT*	CA> = *WK>PROT*	ND	Yes
6	81	CA>COD>>*COM, PROT*	CA>>PROT>*COM, COD*	No	No
7	53	CA>COD>>*PROT, COM*	CA>>*COD, PROT, COM*	Yes	No
8	63	CA>>PROT>*COM*	CA>PROT>>*COM*	Yes	Yes
9	69	WK>CA>>*ACCP>PROT>COM, COD*	WK>CA>>*ACCP*	Yes	Yes
10	71	CA>WK> = PROT>*COM*	PROT>CA>*ACCP> = WK*	Yes	Yes
11	42	CA>>Br>*WK>COD>PROT, COM*	CA>>WK>*PROT*	No	Yes
12	58	CA>>PROT	CA>PROT	ND	No
13	70	ACCP>CA>*PROT>COM*	ACCP>>CA>*PROT*	No	Yes
14	58	WK>CA>>*ACCP>PROT*	WK>>CA>*PROT*	ND	Yes
15	76	CA>>WK>*OCP*>*PROT, ACCP*	PROT>CA>*OCP>WK, ACCP*	No	Yes
16	59	ACCP>CA>>*PROT, MAP*	PROT>>ACCP>CA	ND	Yes
17	75	WK>PROT>*ACCP>CA>COD*	WK>ACCP>*PROT>CA*	Yes	Yes
18	35	WK>> CA>*ACCP*>*COM, PROT*	CA>WK>>*ACCP, PROT*	No	No
19	77	WK>ACCP>>*CA>PROT*	CA>>PROT>*ACCP, WK*	ND	Yes
20	68	CA>Br>COD>*PROT*>*COM*	CA>Br>>*PROT*	No	Yes
21	72	WK>>CA>*PROT*	CA>WK>>*PROT, ACCP*	ND	Yes
22	74	ACCP>CA>>*WK, PROT*	PROT>ACCP>>*CA*	No	Yes
23	67	WK>CA>*ACCP*>>*COD, PROT*	WK>>CA>*PROT*	No	Yes

Br = brushite, ACCP = amorphous carbonated calcium phosphate, CA = carbapatite (carbonated calcium phosphate), COD = calcium oxalate dihydrate, COM = calcium oxalate monohydrate, OCP = octacalcium phosphate pentahydrate, Prot = proteins, WK = whitlockite. ND: not determined.

**Table 2:** Nature and frequency of main chemical phases in 23 prostatic stones.

Chemical phase	Core phase (%)	Main core phase (%)	Peripheral phase (%)	Main peripheral phase (%)
Carbapatite	21 (91%)	12 (52%)	17 (68%)	14 (62%)
Whitlockite	11 (48%)	8 (35%)	7 (30%)	4 (17%)
Amorphous carbonatedcalcium phosphate	6 (26%)	3 (13%)	5 (22%)	1 (4%)
Octacalcium phosphatepentahydrate	1 (4%)	0	2 (9%)	0
Brushite	2 (9%)	0	1 (4%)	0
Proteins	4 (17%)	0	8 (35%)	4 (17%)

## Results

Table 1 summarizes the age of patients, the chemical components of the 23 samples in the core and peripheral layers (main phases [>5%], minor phase [<5%]), the clinical data regarding urinary tract and prostate infection, and the presence of bacterial imprints seen by SEM. Several chemical phases (9 phases) were identified by FTIR spectroscopy. All stones mainly consisted of calcium phosphate, with CA the most common. Interestingly, the frequency of 2 phases, namely whitlockite and amorphous carbonated calcium phosphate (ACCP), as the main components was higher but not significantly in the core than in peripheral layers (48% vs 21%, p = 0.07). SEM revealed bacterial imprints in 18 stones (78%, p = 0.001), but clinical signs of urinary tract infection were reported for only 6 patients.

**Figure 1:**
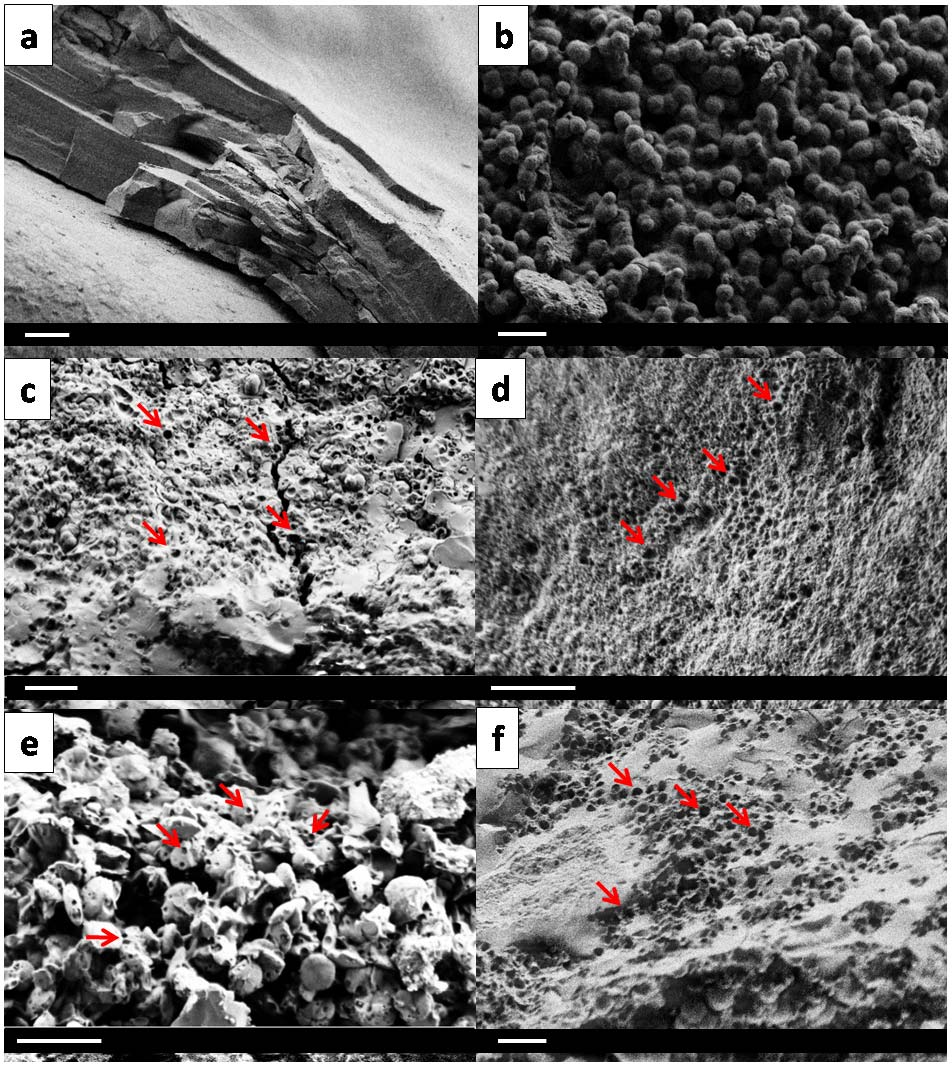
Fixed-effects SEM of prostatic stones. Red arrows show bacterial imprints. a) prostatic carbapatite-amorphous carbonated calcium phosphate (CA-ACCP) stone without any visible imprint; b) spherical prostatic CA stones without any visible imprint; c) prostatic CA-ACCP stone; d) prostatic ACCP stone, e) prostatic CA stone; f) prostatic CA-whitlockite stone. Bar, 4 µm.

Table 2 shows the proportion and frequencies of the main chemical phases in the 23 stones. Whitlockite was detected in the core of 11 stones (48%) and as the main component in 8 (35%) but was the main component of the stone surface in only 4 stones (17%). ACCP was identified in the core of 6 stones (26%) and was the main phase in 3 (13%). ACCP was the main component of the stone surface in only 1 stone (4%).

Representative SEM images of normal stones and those with bacterial imprints (size about 500 nm) on the surface and in the core of stones are in Figure 1.

## Discussion

Our SEM study revealed a high occurrence of bacterial imprints (78%) in 23 prostatic stones, which reveals a past or present infection of the prostate tissue; however, urinary tract infection was detected in only 6 (26%) cases. The large difference between number of reported infections and markers of infection within stones implies that aging may not be the only cause of prostatic calcifications. Infection and a lithogenic process induced by infection may play a role in most of the 99% of surgically removed prostate-containing stones.

The bacterial imprints had a specific spherical shape, which suggests infection by cocci germs. More precisely, the grape-like clustering, shape and size are common with staphylococci infection [18]. In 2 cases of proven infection, the species was *Staphylococcus aureus*. Nevertheless, these imprints were not seen on all stone surfaces. Similarly to kidney stones, the bacteria can imprint on a particle surface such as CA or ACCP but not other crystal types such as whitlockite, octacalcium phosphate or brushite. Indeed, the size of CA and ACCP crystals is smaller than that of other phases, such as struvite [19]. As well, SEM examination was restricted to some parts of small and partial samples collected during prostate removal. However, all stones contained at least 15% CA or ACCP, so a careful observation by SEM allows for detection of bacterial imprints on the surface of these minerals.

We discuss only the major phases. As previously reported, the main compounds of prostatic stones are calcium phosphate [20]. The most common and abundant phase is CA, well known as a common form of ectopic calcification in the kidney [21], vascular system [22], [23] or breast [24]. Particular crystalline phases, namely whitlockite and ACCP, a marker of infection stones in the urinary tract, were identified in the core of 17 (74%) prostatic stones. Whitlockite is an infrequent component of kidney stones and has been associated with chronic urinary tract infection in most calculi from women [8]. This phase has been also found in infections such as tuberculosis [9]. These different markers (imprints, specific phases, etc.) led to the conclusion that 83% of calculi (19 stones, p<0.0001) could be linked to an infection process.

The deleterious consequences of chronic infection and inflammation have been well described in the cellular model [25] and chemical model [26]. A number of papers have highlighted the relation between infection-related inflammation and cancer in various organs such as stomach, liver, lung, colon or bladder [27], [28]. The same reasoning may be applied to prostatic stones. Moreover, recent studies suggest epidemiological and pathologic links between benign prostate hypertrophy and prostate cancer [29]. Chronic infection, as well as the resulting stones, may induce persistent inflammation and could contribute to prostatic hypertrophy. In fact, the inflammatory process, associated with tumor phenomena, seems to influence the formation and evolution of these concretions [30]. We previously reported a high content of proteins in prostate stones [7]. Lactoferrin was found among proteins identified in both corpora amylacea and stones. This protein is considered a marker of inflammation and infiltration by neutrophil polynuclear factors and is implicated in the cancerization process [30].

The clinical interest of this paper is to draw attention to the high occurrence of asymptomatic infection of the prostate.

History of urinary tract infection and risk of renal cell carcinoma have been found to be related. As reported by Parker et al. [31], analysis of epidemiological data suggests a positive association of history of urinary tract infection and renal cell carcinoma development. Similar results were reported by MacLaughin et al. [32] and by Meares for prostate tissue [6]. Clearly more research is needed to establish a relationship because intratissular infection necessitates antibiotic treatment during several weeks. Our data suggest that the presence of stones or calcifications within the prostate could indicate chronic, often asymptomatic infection, the consequence of which remains to be assessed for medical management.

### Conclusions

Prostatic stones are often considered to have no clinical significance, but the use of SEM showed for the first time the high frequency of bacterial imprints in these stones. Moreover, our data underline the specific chemistry of calcium phosphate phases, particularly the preponderance of whitlockite and ACCP in these calcifications. These results demonstrate the high occurence of bacterial infections in the prostate, often without any clinical symptoms.

Inflammation induced by an infection may lead to cancerization of the tissue. Early detection of prostatic calcifications or stones could suggest a search for asymptomatic chronic infection. If an infection is detected, medical management and antibiotic treatment could avoid chronic inflammation of the tissue and further deleterious consequences. Thus, we suggest that discovery of prostate calcifications by imaging such as computerized tomodensitometry might warrant further investigations and management to search for chronic infection of the prostate gland.
